# Activation of Thiamine Pyrophosphokinase TPK-1 Contributes to 6-PPD Quinone-Induced Immunosuppression by Inhibiting Mitochondrial UPR in *Caenorhabditis elegans*

**DOI:** 10.3390/toxics14070630

**Published:** 2026-07-20

**Authors:** Zhe Wu, Guocheng Hu, Yunhui Li, Dayong Wang

**Affiliations:** 1 Key Laboratory of Environmental Medicine Engineering, Ministry of Education, School of Public Health, Southeast University, Nanjing 210009, China; 2 South China Institute of Environmental Sciences, Ministry of Ecology and Environment, Guangzhou 510655, China; 3 Medical School, Southeast University, Nanjing 210009, China

**Keywords:** 6-PPDQ, thiamine pyrophosphate, mt UPR, immunosuppression, nematode

## Abstract

As an emergent contaminant, 6-PPD quinone (6-PPDQ) causes toxic effects in organisms, including induction of immunosuppression. Thiamine pyrophosphate (TPP) serves as an important cofactor for some metabolisms. We aimed to examine the role of thiamine pyrophosphokinase TPK-1 in modulating 6-PPD-induced immunosuppression. Using *Caenorhabditis elegans* as the animal model, 6-PPDQ-exposure treatment was applied to nematodes from the L1 larval stage to adult day 3, and innate immune response was assessed by the expression of antimicrobial genes. In nematodes, 6-PPDQ (1–10 μg/L) elevated TPP content, which was due to an increase in the expression of *tpk-1*, and RNAi of *tpk-1* could block 6-PPDQ-induced immunosuppression. Meanwhile, RNAi of *tpk-1* strengthened expressions of mitochondrial UPR (mt UPR) marker genes (*hsp-6* and *hsp-60*) by activating three transcription factor (TF) genes (*atfs-1*, *ubl-5*, and *dve-1*) in 6-PPDQ-exposed nematodes. RNAi of mitochondrial UPR marker genes and three TF genes aggravated 6-PPDQ-induced immunosuppression. TPK-1 acted upstream of these three TF genes to modulate 6-PPDQ-induced immunosuppression. Moreover, RNAi of *tpk-1* inhibited the expression of complex III/IV subunit genes, and RNAi of these complex III/IV subunit genes conferred resistance to 6-PPDQ-induced immunosuppression. Additionally, these complex III/IV subunit genes functioned upstream of TF genes (*atfs-1*, *ubl-5*, and *dve-1*) to regulate 6-PPDQ-induced immunosuppression. Therefore, our data provide a novel basis for 6-PPDQ-induced immunosuppression mediated by activation of TPK-1 and the following mt UPR suppression.

## 1. Introduction

The tire antioxidant N-(1,3-dimethylbutyl)-N′-phenyl-p-phenylenediamine (6-PPD) undergoes ozone-mediated oxidation to form 6-PPD quinone (6-PPDQ) [1,2,3,4,5]. Elevated traffic has led to widespread detection of this pollutant across soil, surface water, air, road dust, and sediments [6,7,8,9,10,11,12]. Tire wear particles mobilized by rainfall transport substantial amounts of 6-PPDQ into adjacent water bodies and soils [13]. In the environment, concentrations of 6-PPDQ typically span from ng/L to tens of μg/L [14,15]. For example, 6-PPDQ concentrations in road stormwater runoff from the Greater Bay Area were detected at 1.6–94 ng/L [16], while 6-PPDQ concentrations in tunnel wash water reached up to 27 μg/L [17]. Moreover, 6-PPDQ exhibits a pronounced bioaccumulation and multiple aspects of toxicity in organisms, and the bioaccumulation may biomagnify the 6-PPDQ toxicity through food webs [18,19,20,21,22,23,24,25,26,27,28,29]. To date, this pollutant has been detected in fish and mammals, as well as in human urine, blood, and cerebrospinal fluid [30,31], suggesting its exposure risk to environmental animals and human health.

*Caenorhabditis elegans*, a well-established model animal, is helpful for evaluating pollutant toxicity at environmentally relevant concentrations (ERCs) [32,33,34,35,36,37]. Exposure to 6-PPDQ induced some adverse effects in nematodes, including damage on organs and lifespan reduction [38,39,40]. 6-PPDQ caused immunosuppression as evidenced by the markedly reduced expression of *C. elegans* antimicrobial genes, which was linked to further toxicity induction, such as lifespan reduction [41,42]. Meanwhile, some *C. elegans* metabolisms, such as amino acid metabolism, were disrupted by 6-PPDQ exposure [43,44,45]. However, the metabolic basis for 6-PPDQ-caused immunosuppression needs to be further determined.

**Figure 1:**
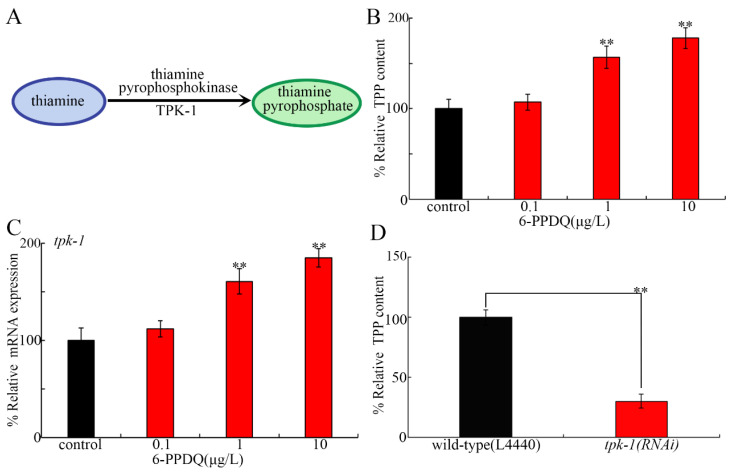
Effect of 6-PPDQ exposure on the expression of *tpk-1* governing TPP synthesis. (**A**) A diagram showing TPP synthesis in nematodes. (**B**) Effect of 6-PPDQ exposure on TPP content. ** *p* < 0.01 vs. the control. (**C**) Effect of 6-PPDQ exposure on the expression of *tpk-1*. ** *p* < 0.01 vs. the control. (**D**) Effect of RNAi of *tpk-1* on TPP content in 6-PPDQ-exposed nematodes. The exposure concentration of 6-PPDQ was 10 μg/L. ** *p* < 0.01.

Thiamine pyrophosphate (TPP) is the bioactive form of vitamin B1 [46], and its synthesis depends on thiamine pyrophosphokinase TPK-1 (Figure 1A) [47]. In *C. elegans*, *tpk-1* mutant is classified into the *clk* mutants that show a long lifespan phenotype [47]. The *clk-1* encodes a coenzyme Q biosynthesis enzyme [48], and RNAi of *clk-1* cause resistance to 6-PPDQ toxicity by activating mitochondrial unfolded protein response (mt UPR) [49,50]. Mitochondrial homeostasis is essential for maintaining the normal physiological state of nematodes [51,52]. Given that mitochondria is a major target of 6-PPDQ [53] and mitochondria plays a crucial role in controlling immunity [54,55], we hypothesize that 6-PPDQ exposure may dysregulate TPK-1 to mediate immunosuppression induction by inhibiting mt UPR. The research aim in the current study was to examine the role of TPK-1 in regulating the immunosuppression induction in 6-PPDQ-exposed nematodes. We thus first investigated the effect of 6-PPDQ on TPP synthesis and *tpk-1* expression. Moreover, the molecular basis for TPK-1 in regulating 6-PPDQ-caused immunosuppression by modulating mt UPR was examined.

## 2. Materials and Methods

### 2.1. Animal Maintenance

*C. elegans* strains were cultured at 20 °C on nematode growth medium (NGM) plates with *E. coli* OP50 as food [56]. Age-synchronized L1 larvae were obtained by standard bleaching and overnight incubation [57]. Genotypes of the strains are described in Table S1.

### 2.2. Exposure

6-PPDQ was dissolved in DMSO to prepare a 1 g/L stock, followed by serial dilution with K buffer to obtain working concentrations (0.1–10 μg/L). These concentrations fall within ERCs of 6-PPDQ [14]. Exposure was performed in L1 larvae and lasted until day 3 of adulthood [58]. During the exposure, 6-PPDQ solutions were refreshed daily, and OP50 was supplied to satisfy larval development.

### 2.3. TPP Content

To quantify TPP levels, a fluorescence-assisted test kit (Tianjingsha Biotech., Suzhou, China) was utilized. Under alkaline conditions, TPP is oxidized to a fluorescent compound, and fluorescence intensity is proportional to the concentration of TPP. Following the manufacturer’s instructions, the nematodes were weighted and homogenized. After centrifugation, supernatants were used for recording fluorescence data (excitation/emission: 365/435 nm) after a 10 min dark incubation. Experiments were repeated three times.

### 2.4. Transcriptional Expression

Nematodes from each experimental group were treated with pre-chilled TRIzol reagent. Total RNA was extracted, and first-strand cDNA was produced using M-MuLV reverse transcriptase. Quantitative real-time polymerase chain reaction (qRT-PCR) was run on a StepOne system with SYBR Green Master Mix. The *tba-1* gene served as the internal control [59]. Each sample was analyzed in triplicate. Primer sequences are detailed in Table S2.

### 2.5. RNA Interference (RNAi)

RNAi was conducted by feeding L1 larval nematodes with *E. coli* HT115 strains producing dsRNA targeting to certain genes, with empty vector L4440 as the control [60]. Progeny were collected for subsequent 6-PPDQ exposure. The efficiency of RNAi was assessed by qRT-PCR (Figures S1 and S2).

### 2.6. Fluorescence Image Assay

CF2018 was used to observe LYS-7::RFP expression [14], and SJ4100 was employed to visualize mt UPR response [61]. After exposure, 50 nematodes were mounted on 2% agarose pads containing 4% formaldehyde, and RFP or GFP images were captured and quantified using ImageJ (version 1.54s).

### 2.7. Lifespan

Lifespan was assessed as described in [62]. Following 6-PPDQ exposure, 50 nematodes from each treatment group were placed onto fresh NGM plates. Survival was scored at 24 h intervals, with death defined as failure to respond to gentle head touch. The median lifespan was determined as the 50% survival time point. Three independent biological replicates were performed.

### 2.8. Mitochondrial Function Assay

Oxygen consumption rate (OCR) and ATP content were assessed to reflect mitochondrial function. The 100 mg nematodes from each group were homogenized. Mitochondria were isolated by differential centrifugation. For OCR detection, 100 μL of mitochondrial suspension was incubated with 4 μL of BBoxiProbe R01 probe (Sangon), and absorbance at 468 nm was recorded every 5 min for 30 min. For the ATP assay, the supernatants were analyzed using an ATP assay kit (Sangon, Shanghai, China). Absorbance was measured at 340 nm, and background ATP was determined using ATP detection working solution. The experiments were repeated three times.

### 2.9. Pseudomonas aeruginosa PA14 Infection and Body Burden

The L4-larvae were infected with *P. aeruginosa* PA14 [63]. *P. aeruginosa* PA14 was seeded on killing plates containing a modified NGM (0.35% instead of 0.25% peptone). *P. aeruginosa* PA14 infection was started by adding nematodes to each plate at 25 °C. Full-lawn PA14 killing plates were prepared for *P. aeruginosa* PA14 infection.

Colony-forming units (CFU) of *P. aeruginosa* PA14 were analyzed [64]. After *P. aeruginosa* PA14 infection for 24 h, nematodes were placed on NGM plates containing ampicillin (1 mg/mL) and gentamicin (1 mg/mL) for 15 min in order to eliminate *P. aeruginosa* PA14 stuck to the body. After that, nematodes were lysed with a motorized pestle, and the lysates were serially diluted with M9 buffer and plated on Luria–Bertani plates containing rifampicin (100 μg/mL) for the selection of *P. aeruginosa* PA14. After incubation at 37 °C overnight, colonies of *P. aeruginosa* PA14 were counted to determine the CFU per nematode. Ten nematodes were examined per treatment, and three replicates were performed.

### 2.10. Data Analysis

Data were presented as mean ± standard derivation (SD). Statistical analysis was conducted using GraphPad Prism (v8). For each dataset, normality was assessed using the Shapiro–Wilk test. Normally distributed two-group comparisons were analyzed by unpaired two-tailed Student’s *t*-test; non-normally distributed two-group comparisons were analyzed by Mann–Whitney U test. For comparisons involving more than two groups, normally distributed data were analyzed by one-way ANOVA followed by Dunnett’s post hoc test, whereas non-normally distributed data were analyzed by the Kruskal–Wallis test followed by Dunn’s multiple-comparison test. A *p* value of <0.01 (**) indicates statistical significance.

## 3. Results

### 3.1. 6-PPDQ Increased TPP Content and tpk-1 Expression

After exposure to 1–10 μg/L 6-PPDQ, a significant elevation in TPP content was observed (Figure 1B). Concurrently, *tpk-1* expression was upregulated by 1–10 μg/L 6-PPDQ exposure (Figure 1C). RNAi of *tpk-1* further led to a reduction in TPP content after 6-PPDQ exposure (Figure 1D).

### 3.2. RNAi of tpk-1 Alleviated 6-PPDQ-Caused Immunosuppression

**Figure 2:**
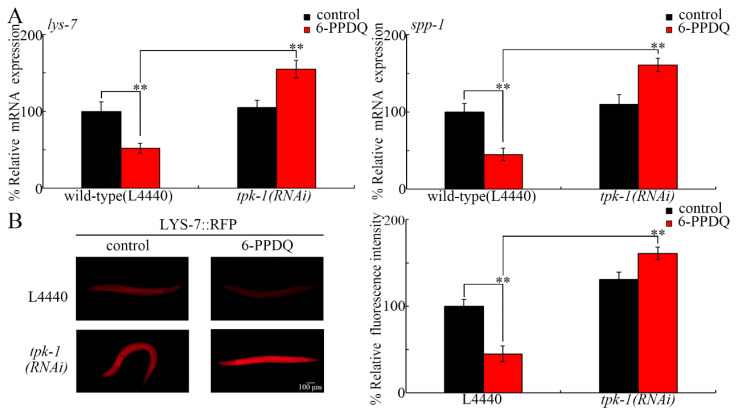
Effect of RNAi of *tpk-1* on 6-PPDQ-caused immunosuppression. (**A**) Effect of RNAi of *tpk-1* on the expressions of *lys-7* and *spp-1* in 6-PPDQ-exposed nematodes. (**B**) Effect of RNAi of *tpk-1* on LYS-7::RFP expression in 6-PPDQ-exposed nematodes. The exposure concentration of 6-PPDQ was 10 μg/L. ** *p <* 0.01.

Exposure to 6-PPDQ (10 μg/L) induced immunosuppression indicated by marked downregulation in expressions of *lys-7*, *spp-1*, and LYS-7::RFP (Figure 2A,B). We next examined the role of TPK-1 in regulating 6-PPDQ-caused immunosuppression. Notably, the inhibitory effect of 6-PPDQ on the expressions of *lys-7*, *spp-1*, and LYS-7::RFP was blocked by *tpk-1* RNAi (Figure 2A,B).

### 3.3. RNAi of tpk-1 Activated mt UPR in 6-PPDQ-Exposed Nematodes

**Figure 3:**
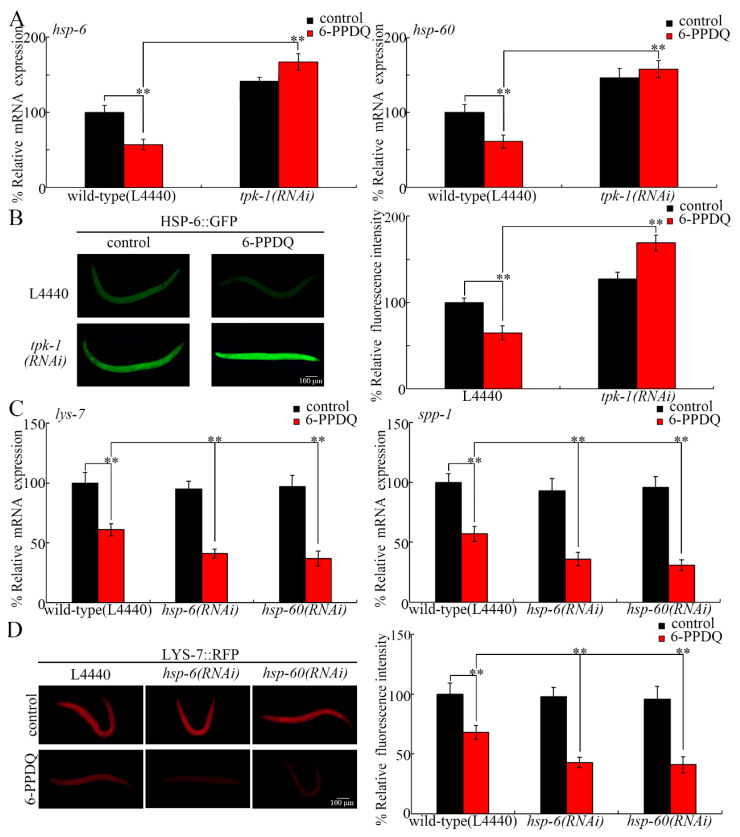
Effect of RNAi of *tpk-1* on mitochondrial UPR in 6-PPDQ-exposed nematodes. (**A**) Effect of RNAi of *tpk-1* on *hsp-6* and *hsp-60* expressions in 6-PPDQ-exposed nematodes. (**B**) Effect of RNAi of *tpk-1* on HSP-6::GFP expression in 6-PPDQ-exposed nematodes. (**C**) Effect of RNAi of *hsp-6* and *hsp-60* on the expressions of *lys-7* and *spp-1* in 6-PPDQ-exposed nematodes. (**D**) Effect of RNAi of *hsp-6* and *hsp-60* on LYS-7::RFP expression in 6-PPDQ-exposed nematodes. The exposure concentration of 6-PPDQ was 10 μg/L. ** *p* < 0.01.

HSP-6 and HSP-60 are markers for monitoring mt UPR activation [65]. 6-PPDQ (10 μg/L) induced suppression in mt UPR, which was indicated by decreased expressions of *hsp-6*, *hsp-60*, and HSP-6::GFP (Figure 3A,B) [66]. Under both the normal condition and the 6-PPDQ exposure condition, RNAi of *tpk-1* increased expressions of *hsp-6*, *hsp-60*, and HSP-6::GFP (Figure 3A,B).

### 3.4. Suppression in mt UPR Mediated 6-PPDQ-Caused Immunosuppression

To examine the association between mt UPR suppression and 6-PPDQ-caused immunosuppression, we assessed the effect of *hsp-6* and *hsp-60* RNAi on 6-PPDQ-caused immunosuppression. The 6-PPDQ-caused decrease in the expressions of *lys-7*, *spp-1*, and LYS-7::RFP was aggravated by *hsp-6* and *hsp-60* RNAi (Figure 3C,D).

### 3.5. RNAi of tpk-1 Activated Transcription Factor Genes Governing mt UPR in 6-PPDQ-Exposed Nematodes

**Figure 4:**
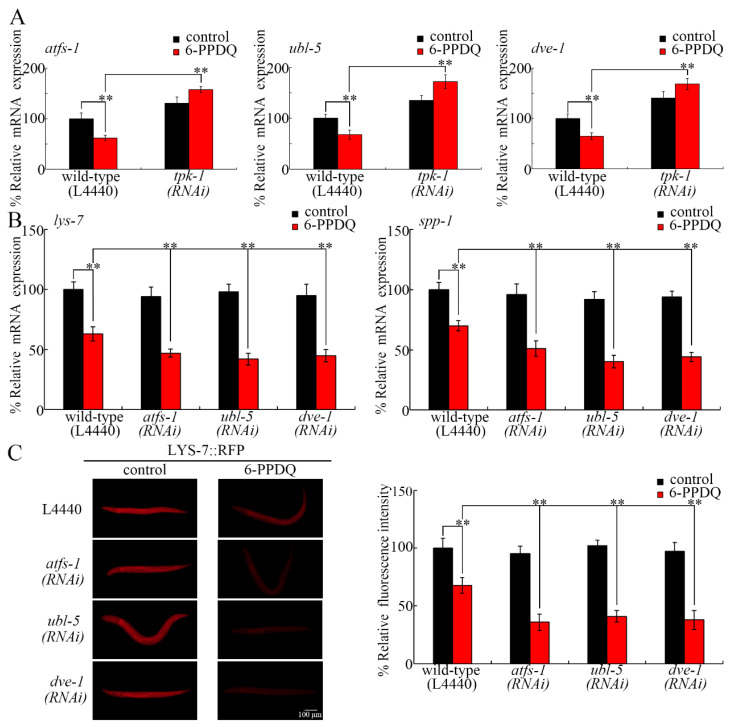
Effect of RNAi of *tpk-1* on the expressions of transcription factor genes governing mt UPR in 6-PPDQ-exposed nematodes. (**A**) Effect of RNAi of *tpk-1* on the expressions of *atfs-1*, *ubl-5*, and *dve-1* in 6-PPDQ-exposed nematodes. (**B**) Effect of RNAi of *atfs-1*, *ubl-5*, and *dve-1* on the expressions of *lys-7* and *spp-1* in 6-PPDQ-exposed nematodes. (**C**) Effect of RNAi of *atfs-1*, *ubl-5*, and *dve-1* on LYS-7::RFP expression in 6-PPDQ-exposed nematodes. The exposure concentration of 6-PPDQ was 10 μg/L. ** *p* < 0.01.

In *C. elegans*, three transcription factors (TFs) (ATFS-1, UBL-5, and DVE-1) were identified to control 6-PPDQ-induced mt UPR suppression [66]. After 6-PPDQ exposure, RNAi of *tpk-1* also upregulated the expression of these three TF genes (Figure 4A). Moreover, the inhibitory effect of 6-PPDQ on the expressions of *lys-7*, *spp-1*, and LYS-7::RFP was further strengthened by RNAi of these three TF genes (Figure 4B,C).

### 3.6. Interaction Between TPK-1 and Transcription Factors in Regulating 6-PPDQ-Caused mt UPR Suppression and Immunosuppression

**Figure 5:**
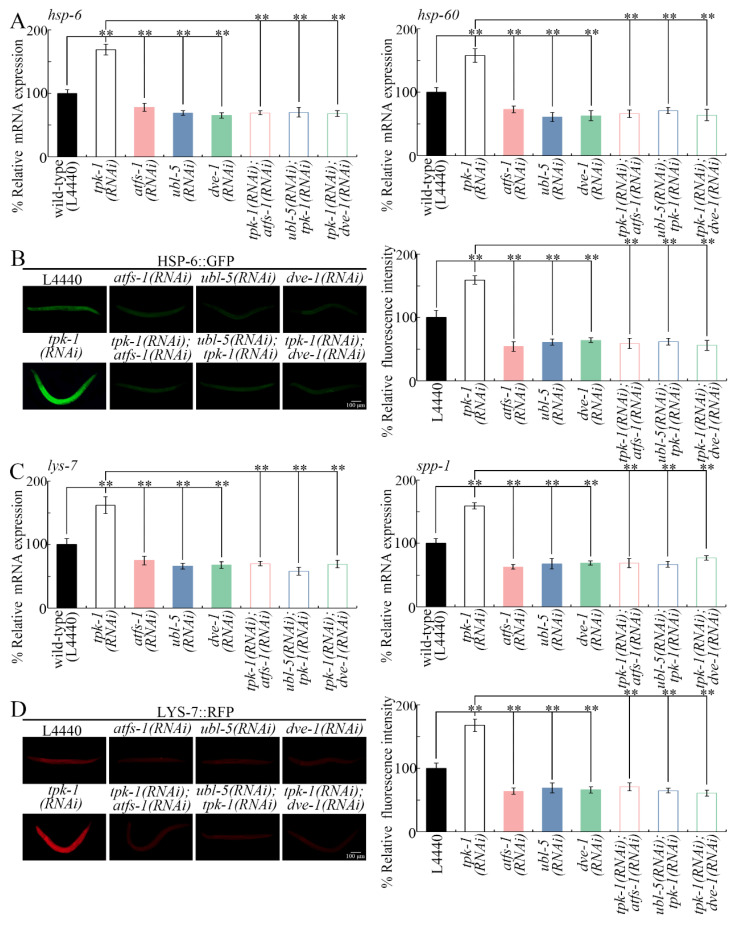
Interaction between *tpk-1* and transcription factor genes in regulating mt UPR and innate immune response in 6-PPDQ-exposed nematodes. (**A**) Interaction between transcription factor genes in regulating expressions of *hsp-6* and *hsp-60* in 6-PPDQ-exposed nematodes. (**B**) Interaction between *tpk-1* and transcription factor genes in regulating HSP-6::GFP expression in 6-PPDQ-exposed nematodes. (**C**) Interaction between *tpk-1* and transcription factor genes in regulating expressions of *lys-7* and *spp-1* in 6-PPDQ-exposed nematodes. (**D**) Interaction between *tpk-1* and transcription factor genes in regulating LYS-7::RFP expression in 6-PPDQ-exposed nematodes. The exposure concentration of 6-PPDQ was 10 μg/L. ** *p* < 0.01.

The analysis of further interactions indicated that the *tpk-1* RNAi-activated expressions of *hsp-6*, *hsp-60*, and HSP-6::GFP were inhibited by RNAi of *atfs-1*, *ubl-5*, and *dve-1* in 6-PPDQ-exposed nematodes (Figure 5A,B). Moreover, after 6-PPDQ exposure, the *tpk-1* RNAi-activated expressions of *lys-7*, *spp-1*, and LYS-7::RFP were also suppressed by RNAi of *atfs-1*, *ubl-5*, and *dve-1* in 6-PPDQ-exposed nematodes (Figure 5C,D).

### 3.7. TPK-1 Modulated Expression of clk-1 and Complex III/IV Subunit Genes in 6-PPDQ-Exposed Nematodes

**Figure 6:**
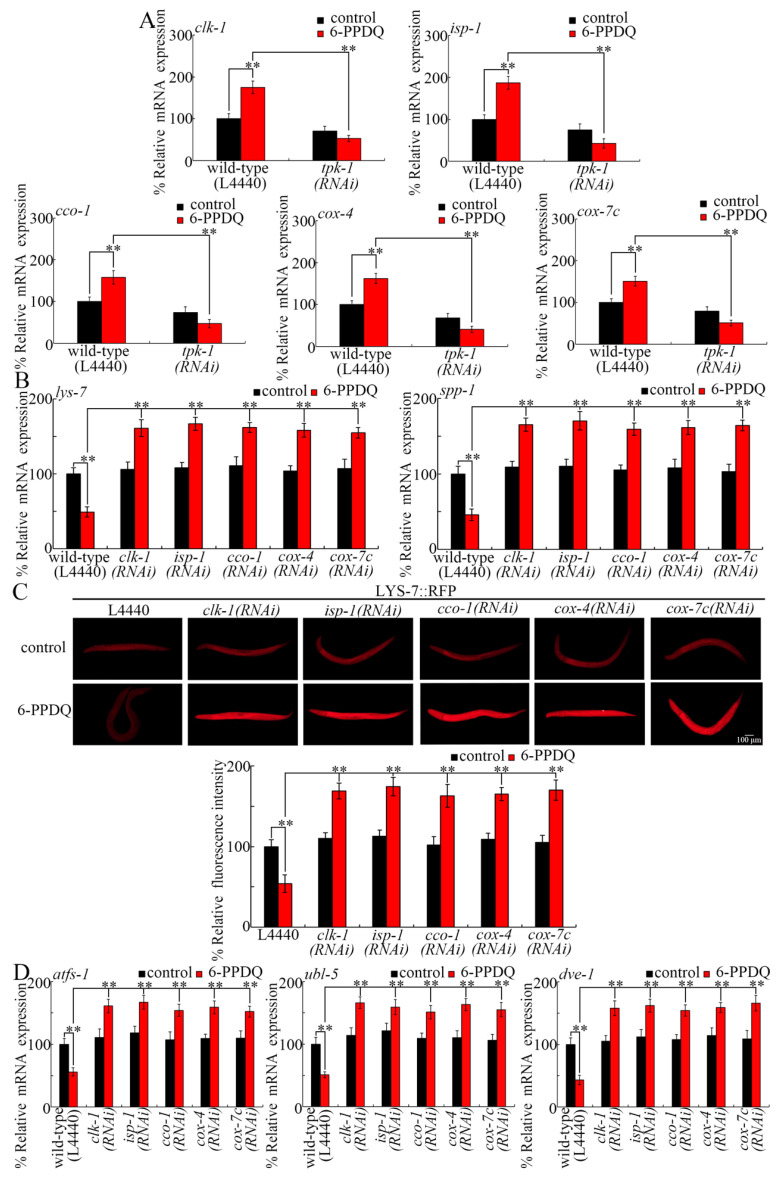
Effect of RNAi of *tpk-1* on the expressions of *clk-1*, *isp-1*, *cco-1*, *cox-4*, and *cox-7c*. (**A**) Effect of RNAi of *tpk-1* on the expressions of *clk-1*, *isp-1*, *cco-1*, *cox-4*, and *cox-7c* in 6-PPDQ-exposed nematodes. (**B**) Effect of RNAi of *clk-1*, *isp-1*, *cco-1*, *cox-4*, and *cox-7c* on the expressions of *lys-7* and *spp-1* in 6-PPDQ-exposed nematodes. (**C**) Effect of RNAi of *clk-1*, *isp-1*, *cco-1*, *cox-4*, and *cox-7c* on LYS-7::RFP expression in 6-PPDQ-exposed nematodes. (**D**) Effect of RNAi of *clk-1*, *isp-1*, *cco-1*, *cox-4*, and *cox-7c* on the expressions of *atfs-1*, *ubl-5*, and *dve-1* in 6-PPDQ-exposed nematodes. The exposure concentration of 6-PPDQ was 10 μg/L. ** *p* < 0.01.

In *C. elegans*, RNAi of *clk-1* and complex III/IV subunit genes (*isp-1*, *cco-1*, *cox-4*, and *cox-7c*) also activated the mt UPR [49,60]. Expressions of these genes were increased by 6-PPDQ (10 μg/L) (Figure 6A), and RNAi of *tpk-1* downregulated these genes after 6-PPDQ exposure (Figure 6A). Moreover, RNAi of *clk-1*, *isp-1*, *cco-1*, *cox-4*, and *cox-7c* could increase expressions of *lys-7*, *spp-1*, and LYS-7::RFP in 6-PPDQ-exposed nematodes (Figure 6B,C).

### 3.8. Interaction Between ATFS-1/UBL-5/DVE-1 and CLK-1/ISP-1/CCO-1/COX-4/COX-7C in Regulating 6-PPDQ-Caused mt UPR Suppression and Immunosuppression

**Figure 7:**
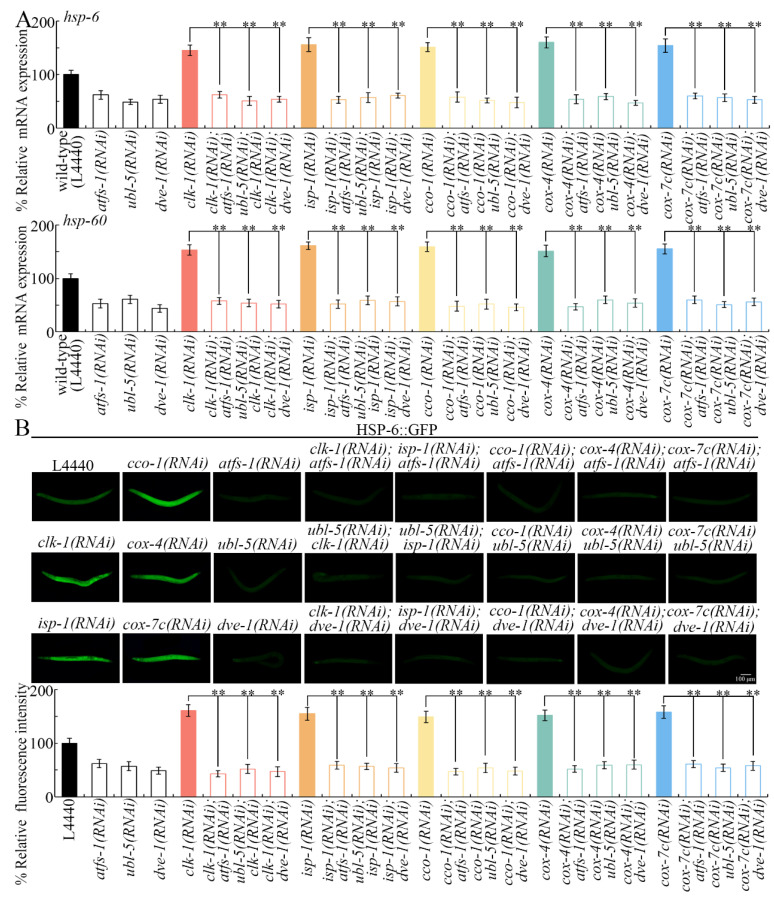
Interaction between *clk-1/isp-1/cco-1/cox-4/cox-7c* and *atfs-1/dve-1/ubl-5* in regulating mt UPR in 6-PPDQ-exposed nematodes. (**A**) Interaction between *clk-1/isp-1/cco-1/cox-4/cox-7c* and *atfs-1/dve-1/ubl-5* in regulating the expressions of *hsp-6* and *hsp-60* in 6-PPDQ-exposed nematodes. (**B**) Interaction between *clk-1/isp-1/cco-1/cox-4/cox-7c* and *atfs-1/dve-1/ubl-5* in regulating HSP-6::GFP expression in 6-PPDQ-exposed nematodes. The exposure concentration of 6-PPDQ was 10 μg/L. ** p < 0.01.

**Figure 8:**
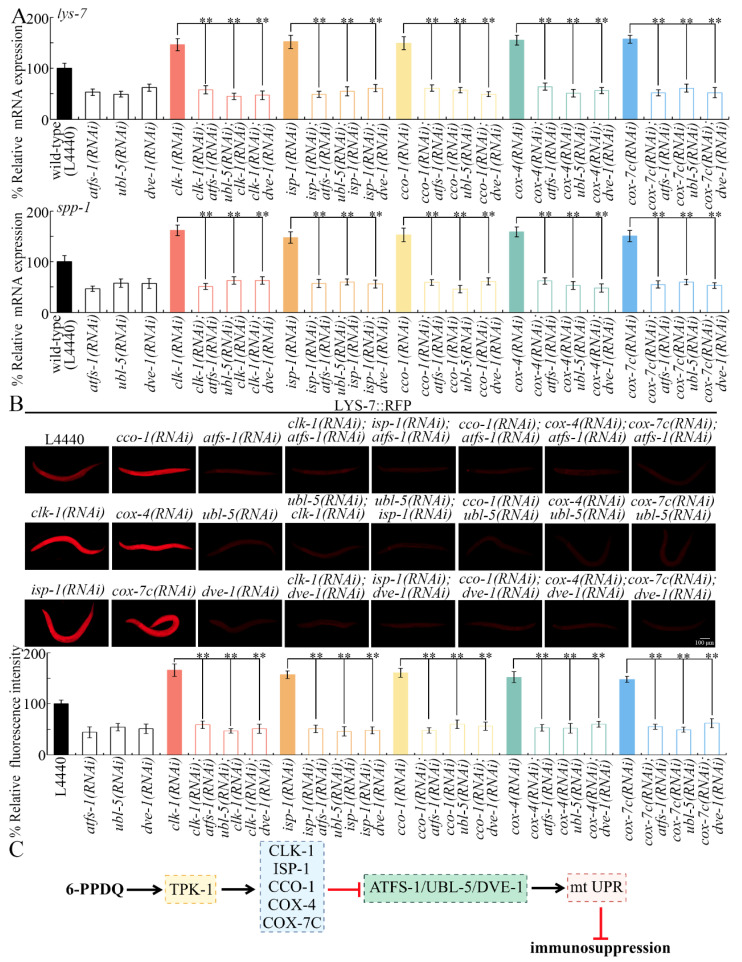
Interaction between *clk-1/isp-1/cco-1/cox-4/cox-7c* and *atfs-1/dve-1/ubl-5* in regulating innate immune response in 6-PPDQ-exposed nematodes. (**A**) Interaction between *clk-1/isp-1/cco-1/cox-4/cox-7c* and *atfs-1/dve-1/ubl-5* in regulating the expressions of *lys-7* and *spp-1* in 6-PPDQ-exposed nematodes. The exposure concentration of 6-PPDQ was 10 μg/L. ** p < 0.01. (**B**) Interaction between *clk-1/isp-1/cco-1/cox-4/cox-7c* and *atfs-1/dve-1/ubl-5* in regulating LYS-7::RFP expression in 6-PPDQ-exposed nematodes. The exposure concentration of 6-PPDQ was 10 μg/L. ** p < 0.01. (**C**) A diagram showing the molecular basis for TPK-1 in regulating 6-PPDQ-caused immunosuppression in nematodes.

After 6-PPDQ exposure, RNAi of *clk-1* and these complex III/IV subunit genes also upregulated the expressions of *atfs-1*, *ubl-5*, and *dve-1* (Figure 6D). Moreover, after 6-PPDQ exposure, activated expressions of *hsp-6*, *hsp-60*, and HSP-6::GFP by RNAi of *clk-1*, *isp-1*, *cco-1*, *cox-4*, and *cox-7c* were inhibited by RNAi of *atfs-1*, *ubl-5*, and *dve-1* (Figure 7A,B). Similarly, after 6-PPDQ exposure, activated expressions of *lys-7*, *spp-1*, and LYS-7::RFP by RNAi of *clk-1*, *isp-1*, *cco-1*, *cox-4*, and *cox-7c* were suppressed by RNAi of *atfs-1*, *ubl-5*, and *dve-1* (Figure 8A,B).

## 4. Discussion

TPK-1 is a *C. elegans* thiamine pyrophosphokinase [47]. Exposure to 6-PPDQ at ERCs increased *tpk-1* expression and elevated the TPP content (Figure 1B,C). RNAi of *tpk-1* suppressed 6-PPDQ-induced TPP accumulation (Figure 1D), which confirms that TPK-1 is a central regulator of TPP homeostasis. Exposure to 6-PPDQ at ERCs could induce immunosuppression indicated by a decrease in antimicrobial genes (*lys-7* and *spp-1*) [41]. Meanwhile, we found the important link between *tpk-1* activation and 6-PPDQ-caused immunosuppression. This is reflected by the observation that RNAi of *tpk-1* effectively reversed the 6-PPDQ-caused downregulation of antimicrobial genes (Figure 2). RNAi of antimicrobial genes (*lys-7* and *spp-1*) strengthened 6-PPDQ-induced lifespan reduction [42], and lifespan can be used as indirect indicator of immunity [67,68]. The 6-PPDQ-induced lifespan reduction was suppressed by *tpk-1* RNAi (Figure S3). RNAi of *tpk-1* also conferred resistance to *P. aeruginosa* PA14 infection and reduced CFU of *P. aeruginosa* PA14 (Figure S4). Recently, DAF-16 and PMK-1 signals were identified to be involved in controlling 6-PPDQ-caused immunosuppression [42]. That is, besides molecular signals, certain metabolism-related signals are possibly also required for controlling 6-PPDQ-caused immunosuppression. These observations imply that TPK-1 activation is not a protective maneuver against immunosuppression; rather, it is the essential metabolic gatekeeper that enables the specific stress signal that causes immunosuppression induced by 6-PPDQ. Recently, we further observed that, after 6-PPDQ exposure, RNAi of *tpk-1* also increase expressions of *dld-1* and *dlst-1* (unpublished data), which are two α-ketoglutarate dehydrogenase complex genes governing the generation of intermediate metabolites on tricarboxylic acid cycle (TCA) [69]. That is, 6-PPDQ inhibits the TCA cycle [69], creating mitochondrial stress. In response, the cell activates TPK-1 to increase TPP, which can meanwhile partially rescue TCA flux.

The mt UPR serves as a conserved surveillance system linking mitochondrial homeostasis to innate immune regulation [70,71]. For the underlying mechanism of TPK-1 function in regulating 6-PPDQ-caused immunosuppression, mt UPR was observed to be activated by RNAi of *tpk-1* after 6-PPDQ exposure (Figure 3A,B). This phenotype of 6-PPDQ-exposed *tpk-1(RNAi)* nematodes was similar to that of 6-PPDQ-exposed nematodes with RNAi of complex III and IV subunit genes [49,60]. Meanwhile, 6-PPDQ-induced mitochondrial dysfunction indicated by an increase in OCR and a decrease in ATP content was also inhibited by RNAi of *tpk-1* (Figure S5). Moreover, several lines of evidence indicate the role of mt UPR activation in mediating the function of *tpk-1* RNAi against 6-PPDQ-caused immunosuppression. First of all, RNAi of mt UPR marker genes (*hsp-6* and/or *hsp-60*) aggravated 6-PPDQ-caused immunosuppression, caused susceptibility to *P. aeruginosa* PA14 infection, and increased CFU of *P. aeruginosa* PA14 (Figure 3C,D and Figure S4). Secondly, *tpk-1* RNAi also increased the expression of TF genes (*atfs-1*, *ubl-5*, and *dve-1*) governing mt UPR activation (Figure 4A), and RNAi of these TF genes enhanced 6-PPDQ-caused immunosuppression and lifespan reduction, which resulted in susceptibility to *P. aeruginosa* PA14 infection, and increased CFU of *P. aeruginosa* PA14 [66] (Figure 4B,C and Figure S4). These TFs establish the core regulators of *C. elegans* mt UPR response [72]. Thirdly, genetic interaction analysis indicated that RNAi of these TF genes suppressed the elevation of mt UPR and the innate immunity caused by *tpk-1* RNAi (Figure 5). Therefore, these data indicate that *tpk-1* RNAi alleviated 6-PPDQ-induced immunosuppression through activating mt UPR. During this process, ATFS-1, UBL-5, and DVE-1 acted downstream of TPK-1 to activate mt UPR and alleviate immunosuppression induced by 6-PPDQ exposure.

Recently, *clk-1* and some complex III and IV subunit genes (*isp-1*, *cco-1*, *cox-4*, and *cox-7c*) were identified to participate in controlling 6-PPDQ toxicity and whose RNAi could activate mt UPR in 6-PPDQ-exposed nematodes [49,60]. More importantly, *clk-1* and these complex III and IV subunit genes were identified as important linkers between *tpk-1* and mt UPR activation during the control of 6-PPDQ-caused immunosuppression. RNAi of *tpk-1* downregulated the expression of *clk-1* and these complex III and IV subunit genes after 6-PPDQ exposure (Figure 5A), and RNAi of *clk-1* and these complex III and IV subunit genes activated both mt UPR and innate immunity and increased lifespan in 6-PPDQ-exposed nematodes (Figure 6B,C) [49,60,66]. Additionally, RNAi of *clk-1* and these complex III and IV subunit genes resulted in resistance to *P. aeruginosa* PA14 infection and decreased CFU of *P. aeruginosa* PA14 (Figure S4). Furthermore, RNAi of *clk-1* and these complex III and IV subunit genes upregulated the expression of TF genes governing mt UPR after 6-PPDQ exposure (Figure 6D), and RNAi of these TF genes could further inhibit mt UPR and innate immunity activated by *clk-1* and complex III and IV subunit genes in 6-PPDQ-exposed nematodes (Figure 7 and Figure 8). Therefore, *clk-1* and complex III and IV subunit genes acted upstream of TF genes to medaite the function of *tpk-1* in regulating 6-PPDQ-caused immunosuppression. That is, a signaling cascade of TPK-1- CLK-1/complex III and IV subunits–TFs was identified to be involved in controlling 6-PPDQ-induced mt UPR suppression and immunosuppression.

## 5. Conclusions

In this study, we observed the activation of thiamine pyrophosphokinase TPK-1 by 6-PPDQ at ERCs, which was accompanied by the elevation of TPP content. RNAi of *tpk-1* prevented 6-PPDQ-caused immunosuppression, which suggests that TPK-1 activation mediated immunosuppression induced by 6-PPDQ. Moreover, TPK-1 regulated 6-PPDQ-caused immunosuppression by enhancing mt UPR driven by three TF genes (*atfs-1*, *ubl-5*, and *dve-1*). Moreover, a signaling cascade of complex III/IV subunit genes–TF genes was identified to mediate the function of TPK-1 in modulating 6-PPDQ-caused immunosuppression through affecting mt UPR (Figure 8C). Our findings suggest the crucial role of TPK-1 activation in mediating 6-PPDQ-caused immunosuppression via modulating mt UPR. That is, this study establishes thiamine pyrophosphokinase TPK-1 activation as a novel mediator of 6-PPDQ-induced immunosuppression through the enhancement of mt UPR via a signaling cascade involving complex III/IV subunit genes and three TF genes (*atfs-1*, *ubl-5*, and *dve-1*). This implies a broader metazoan metabolic control framework in which a thiamine-dependent mitochondrial signaling axis can integrate metabolic disruption with immunosuppression induced by 6-PPDQ, which is suggested to be further confirmed in mammals.

## Data Availability

The original contributions presented in this study are included in the article/Supplementary Materials. Further inquiries can be directed to the corresponding authors.

## Supplementary Materials

The following supporting information can be downloaded at: https://www.mdpi.com/article/10.3390/toxics14070630/s1, Figure S1: RNAi efficiency of *tpk-1*, *hsp-6*, *hsp-60*, *atfs-1*, *ubl-5*, *dve-1*, *clk-1*, *isp-1*, *cco-1*, *cox-4*, and *cox-7c*. ** *p* < 0.01 *vs* wild-type (L4440); Figure S2: RNAi efficiency of genes in nematodes with double RNAi of certain genes. ** *p* < 0.01 *vs* wild-type (L4440); Figure S3: Effect of RNAi of *tpk-1* on lifespan in 6-PPDQ exposed nematodes. Exposure concentration of 6-PPDQ was 10 μg/L. ** *p* < 0.01; Figure S4: Effect of RNAi of *tpk-1*, *clk-1*, *isp-1*, *cco-1*, *cox-4*, *cox-7c*, *atfs-1*, *ubl-5*, *dve-1*, and *hsp-6* on survival of nematodes infected with *P. aeruginosa*. PA14 (A) and CFU of *P. aeruginosa* PA14 (B). The 6-PPDQ exposure concentration was 10 μg/L. ** *p* < 0.01; Figure S5: Effect of RNAi of *tpk-1* on OCR (A) and ATP content (B) in 6-PPDQ exposed nematodes. The 6-PPDQ exposure concentration was 10 μg/L. ** *p* < 0.01. Table S1: Strain information; Table S2: Primer information for qRT-PCR.

## References

[1] Hua X., Wang D.-Y. (2023). Tire-rubber related pollutant 6-PPD quinone: A review of its transformation, environmental distribution, bioavailability, and toxicity. J. Hazard. Mater..

[2] Jin L., Cheng S., Ge M., Ji L. (2024). Evidence for the formation of 6PPD-quinone from antioxidant 6PPD by cytochrome P450. J. Hazard. Mater..

[3] Bohara K., Timilsina A., Adhikari K., Kafle A., Basyal S., Joshi P., Yadav A.K. (2024). A mini review on 6PPD quinone: A new threat to aquaculture and fisheries. Environ. Pollut..

[4] Platt K.L., Yushchenko O., Laszakovits J.R., Zhang Y., Pflug N.C., McNeill K. (2025). Aquatic thermal and photochemical reactivity of N-(1,3-dimethylbutyl)-N’-phenyl-p-phenylenediamine (6PPD), N-Isopropyl-N’-phenyl-p-phenylenediamine (IPPD), and 6PPD-quinone. Environ. Sci. Technol..

[5] Wang S., Xu S., Lu J., Wan Y., Kang Z., Chen H., Islam S., Gao B. (2026). Occurrence, transformation, and toxicity of tire-derived chemicals 6PPD and 6PPD-q in the environment. Environ. Sci. Technol..

[6] Liang Y., Zhu F., Li J., Wan X., Ge Y., Liang G., Zhou Y. (2024). p-Phenylenediamine antioxidants and their quinone derivatives: A review of their environmental occurrence, accessibility, potential toxicity, and human exposure. Sci. Total Environ..

[7] Li Y., Zeng J., Liang Y., Zhao Y., Zhang S., Chen Z., Zhang J., Shen X., Wang J., Zhang Y. (2024). A review of N-(1,3-dimethylbutyl)-N’-phenyl-p-phenylenediamine (6PPD) and its derivative 6PPD-quinone in the environment. Toxics.

[8] Hiki K., Yamamoto H. (2022). Concentration and leachability of N-(1,3-dimethylbutyl)-N’-phenyl-p-phenylenediamine (6PPD) and its quinone transformation product (6PPD-Q) in road dust collected in Tokyo, Japan. Environ. Pollut..

[9] Ihenetu S.C., Xu Q., Khan Z.H., Kazmi S.S.U.H., Ding J., Sun Q., Li G. (2024). Environmental fate of tire-rubber related pollutants 6PPD and 6PPD-Q: A review. Environ. Res..

[10] Zhao H.N., Hu X., Tian Z., Gonzalez M., Rideout C.A., Peter K.T., Dodd M.C., Kolodziej E.P. (2023). Transformation products of tire rubber antioxidant 6PPD in heterogeneous gas-phase ozonation: Identification and environmental occurrence. Environ. Sci. Technol..

[11] Seiwert B., Nihemaiti M., Troussier M., Weyrauch S., Reemtsma T. (2022). Abiotic oxidative transformation of 6-PPD and 6-PPD quinone from tires and occurrence of their products in snow from urban roads and in municipal wastewater. Water Res..

[12] Reemtsma T., Seiwert B., Yeomans P., Mumford R., Bennett S. (2026). Abiotic transformation of radiolabelled 6-PPD and 6-PPDQ in water and in presence of nitrogen/air/ozone and light. Ecotoxicol. Environ. Saf..

[13] Shen D., Shi Q., Zhang J., Sy N.D., Yates R., Wang W., Gan J. (2025). Transformations of 6PPD and 6PPD-quinone in soil under redox-driven conditions: Kinetics, product identification, and environmental implications. Environ. Int..

[14] Wan X., Liang G.-Y., Wang D.-Y. (2024). Potential human health risk of the emerging environmental contaminant 6-PPD quinone. Sci. Total Environ..

[15] Yi J., Ruan J., Yu H., Wu B., Zhao J., Wang H., Chen R., Yang Q., Chen J., Sun D. (2025). Environmental fate, toxicity, and mitigation of 6PPD and 6PPD-quinone: Current understanding and future directions. Environ. Pollut..

[16] Liu Y.H., Mei Y.X., Liang X.N., Ge Z.Y., Huang Z., Zhang H.Y., Zhao J.L., Liu A., Shi C., Ying G.G. (2024). Small-intensity rainfall triggers greater contamination of rubber-derived chemicals in road stormwater runoff from various functional areas in megalopolis cities. Environ. Sci. Technol..

[17] Varshney S., O’Connor O.L., Gora A.H., Rehman S., Kiron V., Siriyappagouder P., Dahle D., Kogel T., Ørnsrud R., Olsvik P.A. (2024). Mixture toxicity of 6PPDquinone and polystyrene nanoplastics in zebrafish. Environ. Pollut..

[18] Jiang Y., Wang C., Ma L., Gao T., Wāng Y. (2024). Environmental profiles, hazard identification, and toxicological hallmarks of emerging tire rubber-related contaminants 6PPD and 6PPD-quinone. Environ. Int..

[19] Jiao F., Zhao Y., Yue Q., Wang Q., Li Z., Lin W., Han L., Wei L. (2025). Chronic toxicity mechanisms of 6PPD and 6PPD-Quinone in zebrafish. Environ. Sci. Ecotechnol..

[20] Li R., Barrett H., Nair P., Wang M., Tomlin H., Atkinson J.B., Krogh E., Xie L., Peng H. (2025). Enantioselectivity in metabolism and toxicity of 6PPD-quinone in Salmonids. Environ. Sci. Technol..

[21] Fang L., Xu J., Fang C., Jin Y. (2025). Oral exposure to tire rubber-derived contaminant 6PPD and 6PPD-quinone induces intestinal toxicity in mice. Toxicology.

[22] Yu H., Zhang W., Wang D., Shi B., Zhu Y., Hu W., He J., Hong J., Xu X., Zheng X. (2025). Exposure to 6PPD-Q induces dysfunctions of ovarian granulosa cells: Its potential role in PCOS. J. Hazard. Mater..

[23] Calle L., Le Du-Carrée J., Martínez I., Sarih S., Montero D., Gómez M., Almeda R. (2025). Toxicity of tire rubber-derived pollutants 6PPD-quinone and 4-tert-octylphenol on marine plankton. J. Hazard. Mater..

[24] Wang Q.N., Wang C., Wāng Y. (2026). Inhalation exposure to tire rubber particle-sourced pollutant 6PPD-quinone involving basolateral amygdala impairment in male ICR mice. J. Adv. Res..

[25] Foldvik A., Kryuchkov F., Ulvan E.M., Sandodden R., Kvingedal E. (2024). Acute toxicity testing of Pink Salmon (*Oncorhynchus gorbuscha*) with the tire rubber-derived chemical 6PPD-quinone. Environ. Toxicol. Chem..

[26] Gamil M.R., Abu-Elala N.M., Abo-Al-Ela H.G. (2025). Toxicity of 6PPD-quinone in European seabass (*Dicentrarchus labrax*) under baseline and Vibrio alginolyticus challenge conditions: Protective insights from astaxanthin mitigation. Sci. Total Environ..

[27] Roberts C., Kohlman E., Jain N., Amekor M., Alcaraz A.J., Hogan N., Brinkmann M., Hecker M. (2025). Subchronic and acute toxicity of 6PPD-quinone to earlylife stage Rainbow trout (*Oncorhynchus mykiss*). Environ. Sci. Technol..

[28] He W., Chao J., Gu A., Wang D. (2024). Evaluation of 6-PPD quinone toxicity on lung of male BALB/c **mice** by quantitative proteomics. Sci. Total Environ..

[29] Qiu X.W., Zhang W., Chen X., Luo G., Qi X., Guo Q., Zhou X., Sun X., Xiang H., Feng H. (2025). Respiratory exposure to 6PPD-quinone aggravates Klebsiella pneumoniae pneumonia by impairing the innate immune function of alveolar macrophages. J. Hazard. Mater..

[30] Ma C.S., Li D.L., Wang F., Wang J.P., He M.T. (2024). Neurotoxicity from long-term exposure to 6-PPDQ: Recent advances. Ecotoxicol. Environ. Saf..

[31] Xu F., Su M., Tang S. (2026). Spatiotemporal distribution of 6PPD-Q in China revealed by a national-scale quantification framework. Environ. Sci. Technol..

[32] Wang D.-Y. (2022). Toxicology at Environmentally Relevant Concentrations in Caenorhabditis elegans.

[33] Yao Y., Zhang T., Tang M. (2022). A critical review of advances in reproductive toxicity of common nanomaterials to *Caenorhabditis elegans* and influencing factors. Environ. Pollut..

[34] Chen H., Wang C., Li H., Ma R., Yu Z., Li L., Xiang M., Chen X., Hua X., Yu Y. (2019). A review of toxicity induced by persistent organic pollutants (POPs) and endocrine-disrupting chemicals (EDCs) in the nematode *Caenorhabditis elegans*. J. Environ. Manag..

[35] Wu Z., Wang L., Chen W., Wang Y., Cui K., Chen W., Liu J., Jin H., Zhou Z. (2024). Reproductive toxicity and multi/transgenerational effects of emerging pollutants on *C. elegans*. Toxics.

[36] Hartman J.H., Widmayer S.J., Bergemann C.M., King D.E., Morton K.S., Romersi R.F., Jameson L.E., Leung M.C.K., Andersen E.C., Taubert S. (2021). Xenobiotic metabolism and transport in *Caenorhabditis elegans*. J. Toxicol. Environ. Health B Crit. Rev..

[37] Crombie T.A., Pamminger T., Andersen E.C., Glaberman S. (2026). High-throughput toxicity screening with *C. elegans*: Current platforms, key advantages, and future directions. Environ. Sci. Technol..

[38] Wang W., Hu G.-C., Wang D.-Y. (2026). 6-PPD quinone inhibits ammonia excretion to cause multiple aspects of toxicity in *Caenorhabditis elegans* by activating dual oxidase complex-SKN-1 axis. Environ. Pollut..

[39] Wu J.-W., Bian Q., Wang D.-Y. (2026). 6-PPD quinone inhibits phosphatidic acid synthesis associated with an increase in intestinal barrier permeability in *C. elegans*. Toxics.

[40] Wang Y.-X., Hu G.-C., Wang D.-Y. (2026). 6-PPD quinone reduces lifespan by activating a feedback loop between cholesterol transformation related signal and insulin signaling in *C. elegans*. J. Environ. Sci..

[41] Wang Y.-X., Wang D.-Y. (2024). Transgenerational intestinal toxicity of 6-PPD quinone in causing ROS production, enhancement in intestinal permeability and suppression in innate immunity in *C. elegans*. Environ. Pollut..

[42] Shu C., Wang W., Cao S., Hou B., Gu S., Fu L., Zhang F., Wang D. (2026). 6-PPD quinone induces lifespan reduction by causing immunosuppression via DAF-16/PMK-1 signaling in *Caenorhabditis elegans*. Environ. Chem. Ecotoxicol..

[43] Hu D.-Y., Wang Y.-X., Hu G.-C., Liu R., Wang D.-Y. (2025). 6-PPD quinone inhibited retinoic acid synthesis mediates toxicity through feedback loop between ALH-3/DHS-19-SEX-1 axis and intestinal signals in *Caenorhabditis elegans*. J. Environ. Expo. Assess..

[44] Wang Y.-X., Hu G.-C., Wang D.-Y. (2025). Increased S-adenosyl methionine strengthens the suppression in mitochondrial unfolded protein response induced by 6-PPD quinone at environmentally relevant concentrations in *Caenorhabditis elegans*. Environ. Pollut..

[45] Wang W., Li Y.-H., Wang D.-Y. (2026). Simultaneously PYCR-1 and ALH-6 inhibition exacerbates 6-PPD quinone toxicity via disrupting proline and glutamate metabolisms and activating insulin signals in *Caenorhabditis elegans*. J. Environ. Sci..

[46] Makarchikov A.F., Wins P., Bettendorff L. (2025). Biochemical and medical aspects of vitamin B1 research. Neurochem. Int..

[47] de Jong L., Meng Y., Dent J., Hekimi S. (2004). Thiamine pyrophosphate biosynthesis and transport in the nematode *Caenorhabditis elegans*. Genetics.

[48] Jonassen T., Larsen P.L., Clarke C.F. (2001). A dietary source of coenzyme Q is essential for growth of long-lived *Caenorhabditis elegans clk-1* mutants. Proc. Natl. Acad. Sci. USA.

[49] Hua X., Wang D.-Y. (2025). 6-PPD quinone causes alteration in ubiquinone-mediated complex III associated with toxicity on mitochondrial function and longevity in *Caenorhabditis elegans*. J. Environ. Chem. Eng..

[50] Bong D., Kwon H.C., Lee S. (2026). Multilayered regulation of longevity in *Caenorhabditis elegans*. Mol. Cells.

[51] Le H.T., Yu J., Ahn H.S., Kim M., Chae I.G., Cho H., Kim J., Park H., Kwon H.N., Chae H. (2025). eIF2α phosphorylation-ATF4 axis-mediated transcriptional reprogramming mitigates mitochondrial impairment during ER stress. Mol. Cells.

[52] Anderson N.S., Haynes C.M. (2020). Folding the mitochondrial UPR into the integrated stress response. Trends Cell Biol..

[53] Hua X., Liang G.-Y., Chao J., Wang D.-Y. (2024). Exposure to 6-PPD quinone causes damage on mitochondrial complex I/II associated with lifespan reduction in *Caenorhabditis elegans*. J. Hazard. Mater..

[54] Pellegrino M.W., Nargund A.M., Kirienko N.V., Gillis R., Fiorese C.J., Haynes C.M. (2014). Mitochondrial UPR-regulated innate immunity provides resistance to pathogen infection. Nature.

[55] Bahat A., MacVicar T., Langer T. (2021). Metabolism and innate immunity meet at the mitochondria. Front. Cell Dev. Biol..

[56] Brenner S. (1974). The genetics of *Caenorhabditis elegans*. Genetics.

[57] Wang Y.-X., Wang D.-Y. (2026). Disruption of arginine metabolism by 6-PPD quinone underlies its mitochondrial toxicity at environmentally relevant concentrations in *Caenorhabditis elegans*. J. Environ. Expo. Assess..

[58] Su R., Wang Y.-X., Bian Q., Wang D.-Y. (2026). Exposure risk of 6-PPD quinone in causing immunosuppression: Role of disruption in FAD synthesis and riboflavin transporters. J. Hazard. Mater. Adv..

[59] Wu J.-W., Li L.-E., Hu D.-Y., Liu R., Bian Q., Wang D.-Y. (2025). Environmentally relevant concentrations of 6-PPDQ disrupts vitamin D3 adsorption and receptor function in *Caenorhabditis elegans*. Environ. Sci. Process. Impacts.

[60] Hua X., Wang D.-Y. (2025). 6-PPD quinone at environmentally relevant concentrations activates feedback response of electron transport chain to mediate damage on mitochondrial function and longevity in *Caenorhabditis elegans*. Environ. Chem. Ecotoxicol..

[61] Liu H.-L., Wang D.-Y. (2021). Intestinal mitochondrial unfolded protein response induced by nanoplastic particles in *Caenorhabditis elegans*. Chemosphere.

[62] Hua X., Wang D.-Y. (2026). Exposure to 6-PPD quinone inhibits ATP synthesis by causing damage on mitochondrial complex V in *Caenorhabditis elegans*. Environ. Res..

[63] Yu Y.-L., Zhi L.-T., Wu Q.-L., Jing L.-N., Wang D.-Y. (2018). NPR-9 regulates innate immune response in *Caenorhabditis elegans* by antagonizing activity of AIB interneurons. Cell. Mol. Immunol..

[64] Zhi L.-T., Yu Y.-L., Li X.-Y., Wang D.-Y., Wang D.-Y. (2017). Molecular control of innate immune response to *Pseudomonas aeruginosa* infection by intestinal *let-7* in *Caenorhabditis elegans*. PLoS Pathog..

[65] Wang D.-Y. (2019). Molecular Toxicology in Caenorhabditis elegans.

[66] Hua X., Wang D.-Y. (2024). 6-PPD quinone at environmentally relevant concentrations induced damage on longevity in *C. elegans*: Mechanistic insight from inhibition in mitochondrial UPR response. Sci. Total Environ..

[67] Ermolaeva M.A., Schumacher B. (2014). Insights from the worm: The *C. elegans* model for innate immunity. Semin. Immunol..

[68] Martineau C.N., Kirienko N.V., Pujol N. (2021). Innate immunity in *C. elegans*. Curr. Top. Dev. Biol..

[69] Wan X., Liang G.-Y., Wang D.-Y. (2025). 6-PPD quinone at environmentally relevant concentrations disrupts citric acid cycle in Caenorhabditis elegans: Role of reduction in acetyl CoA and pyruvate contents. Environ. Chem. Ecotoxicol..

[70] Kim S., Ramalho T.R., Haynes C.M. (2024). Regulation of proteostasis and innate immunity via mitochondria-nuclear communication. J. Cell Biol..

[71] Ghosh A., Singh J. (2026). Interplay between proteostasis pathways and innate immune responses in *Caenorhabditis elegans*. Infect. Immun..

[72] Zhou Z., Fan Y., Zong R., Tan K. (2022). The mitochondrial unfolded protein response: A multitasking giant in the fight against human diseases. Ageing Res. Rev..

