# Supplementary material for: Activation of Thiamine Pyrophosphokinase TPK-1 Contributes to 6-PPD Quinone-Induced Immunosuppression by Inhibiting Mitochondrial UPR in Caenorhabditis elegans
Source: Toxics. 2026 Jul 20;14(7):630. doi: 10.3390/toxics14070630 (PMC13417975; doi:10.3390/toxics14070630)
Supplement: Supplementary file 1 [file toxics-14-00630-s001.zip › toxics-4442292-supplementary.pdf]

Supporting Information:

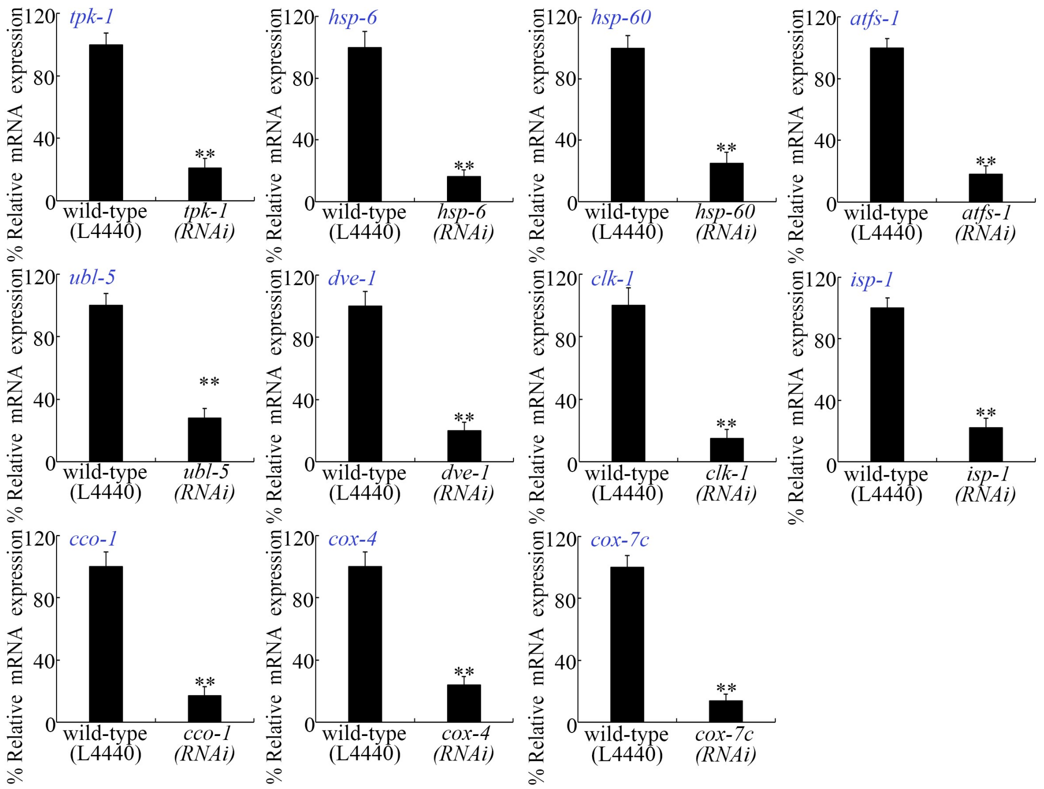

Figure S1. RNAi efficiency of *tpk-1*, *hsp-6*, *hsp-60*, *atfs-1*, *ubl-5*, *dve-1*, *clk-1*, *isp-1*, *cco-1*, *cox-4*, and *cox-7c*. $^{**}p < 0.01$ *vs* wild-type (L4440).

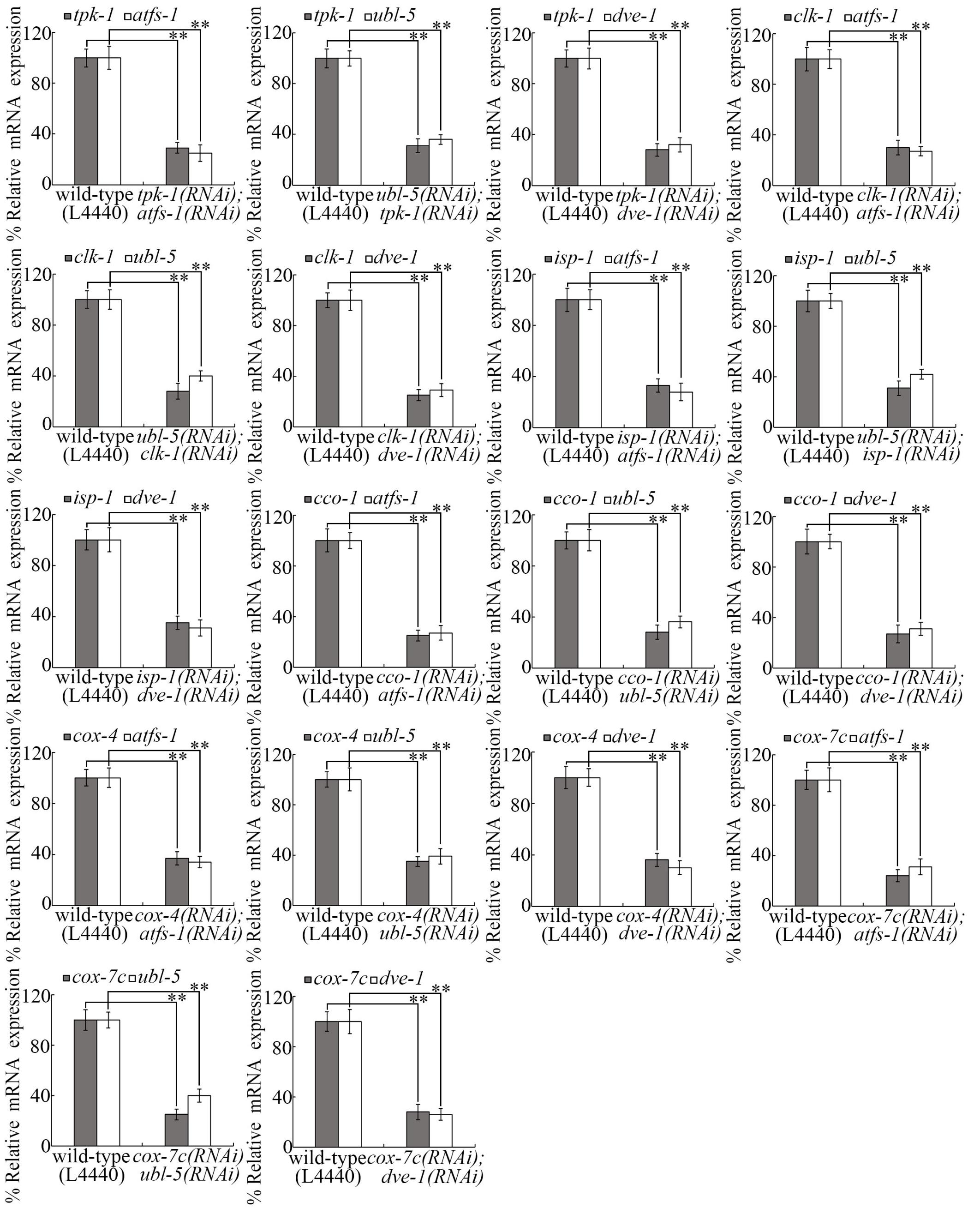

Figure S2. RNAi efficiency of genes in nematodes with double RNAi of certain genes. $^{**}p < 0.01$ *vs* wild-type (L4440).

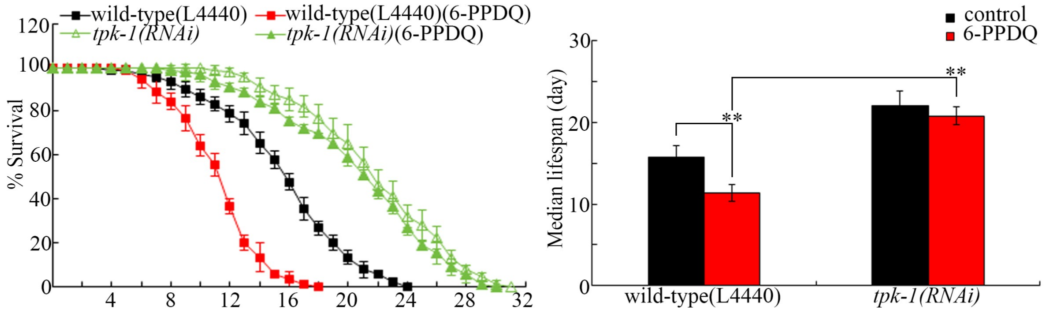

Figure S3. Effect of RNAi of *tpk-1* on lifespan in 6-PPDQ exposed nematodes. Exposure concentration of 6-PPDQ was 10 μg/L. $^{**}p < 0.01$.

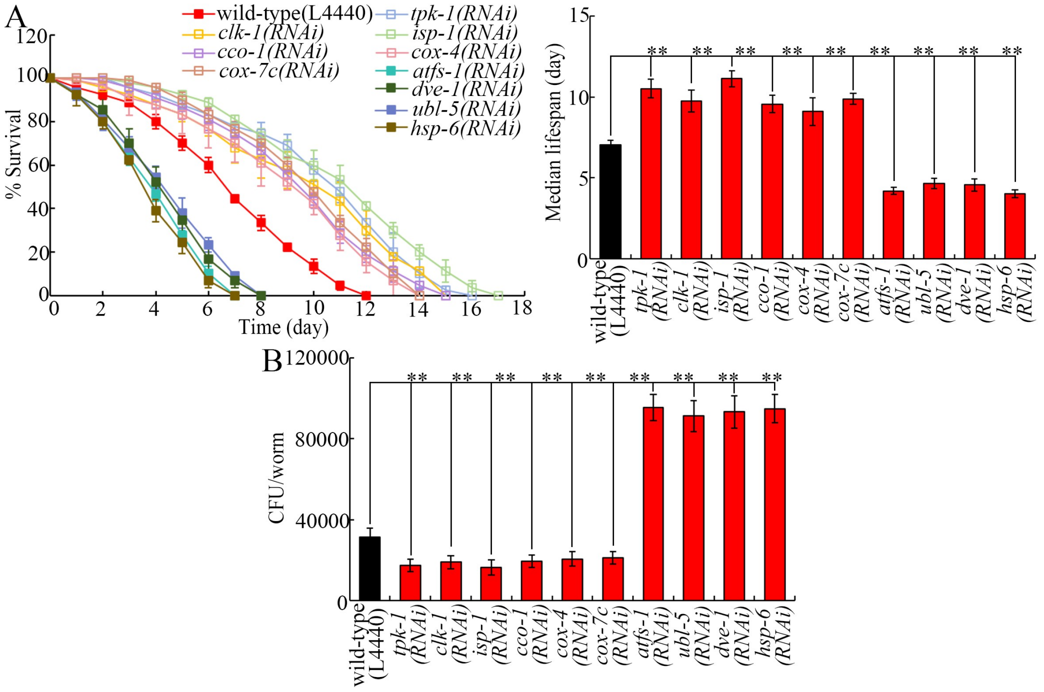

Figure S4. Effect of RNAi of *tpk-1*, *clk-1*, *isp-1*, *cco-1*, *cox-4*, *cox-7c, atfs-1*, *ubl-5*, *dve-1*, and *hsp-6* on survival of nematodes infected with *P. aeruginosa.* PA14 (A) and CFU of *P. aeruginosa* PA14 (B). The 6-PPDQ exposure concentration was 10 μg/L. $^{**}p < 0.01$.

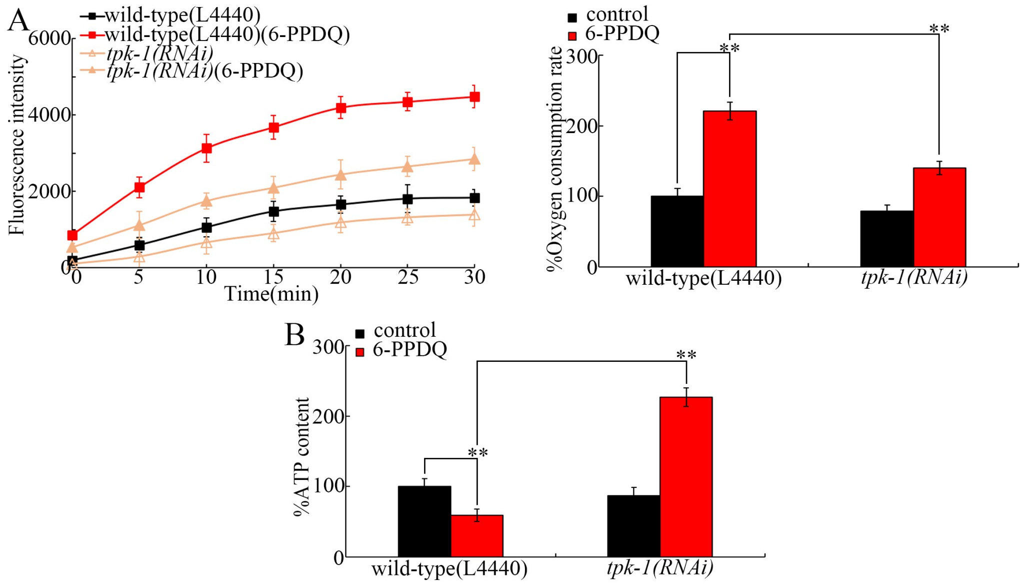

Figure S5. Effect of RNAi of *tpk-1* on OCR (A) and ATP content (B) in 6-PPDQ exposed nematodes. The 6-PPDQ exposure concentration was 10 μg/L. $^{**}p < 0.01$.

Table S1. Strain information.

Strains	Genotype	Description
N2		Wild-type
CF2018	*muEx304[lys-7p::RFP(NLS) + rol-6(su1006)]*	Transgenic strain expressing LYS-7::RFP
SJ4100	*zcIs13[hsp-6p::GFP + lin-15(+)]*	Transgenic strain expressing HSP-6::GFP

Table S2. Primer information for qRT-PCR.

Gene	Forward primer (5'-3')	Reverse primer (5'-3')
tpk-1	CTCGAAGAGCGTTCAGTGGT	ATCCCCTGTAGGAACAGCGA
lys-7	GGTTCCCCCGATTGTTGACT	TGGCGAAGTGACCTGAATCC
spp-1	TGACTCGCATTCTTCCGTGT	GCAACGGCAACAGCATAGTC
hsp-6	ACAGGCCATGCAGAGACTTC	CTTGAACAGTGGCTTGCACC
hsp-60	CGTGGGGAAGCCCAAAGAT	TTCCGACCTTCTTCATGGCG
atfs-1	CAGTTTTGCGCACAGCTTCT	CCATTCTGCCATGAGTCGGT
ubl-5	TGATCGCTGCACAAACTGGA	GCTCGAAATTGAATCCCTCGTG
dve-1	CGTCGAAACTGTACTAGCAGC	CGTTTCCAGCGTATCAAGCC
clk-1	GCTTATGCTCTCGGTGTCGGTTCA	ATCGTCGGCAAGGAGTTCTTTCAA
isp-1	CATTCCCCGATATGTCAAACTATC	TGTACAACTTCCTTTCCGGCCCAC
cco-1	CGTCGTACTTTGGCCACTGA	TTTGGCTTCTGCTTGGTGGA
cox-4	CCGTTATCCTCTCCGTGCTC	AGCGACGACAACCAAGAGAG
cox-7c	CTCTGCTCGTCTCCCATTCC	ACCGTGCTGTTGTACCGTTT
tba-1	TCAACACTGCCATCGCCGCC	TCCAAGCGAGACCAGGCTTCAG
